# Identification of Prostaglandin F2 Receptor Negative Regulator (PTGFRN) as an internalizable target in cancer cells for antibody-drug conjugate development

**DOI:** 10.1371/journal.pone.0246197

**Published:** 2021-01-27

**Authors:** Jorge Marquez, Jianping Dong, Chun Dong, Changsheng Tian, Ginette Serrero

**Affiliations:** 1 Target Discovery Division, A&G Pharmaceutical, Inc., Columbia, Maryland, United States of America; 2 Department of Pharmaceutical Sciences, University of Maryland, Baltimore School of Pharmacy, Baltimore, Maryland, United States of America; 3 Precision Antibody Division, A&G Pharmaceutical, Inc., Columbia, Maryland, United States of America; Fondazione IRCCS Istituto Nazionale dei Tumori, ITALY

## Abstract

Antibody-drug conjugates (ADC) are effective antibody-based therapeutics for hematopoietic and lymphoid tumors. However, there is need to identify new targets for ADCs, particularly for solid tumors and cancers with unmet needs. From a hybridoma library developed against cancer cells, we selected the mouse monoclonal antibody 33B7, which was able to bind to, and internalize, cancer cell lines. This antibody was used for identification of the target by immunoprecipitation and mass spectrometric analysis, followed by target validation. After target validation, 33B7 binding and target positivity were tested by flow cytometry and western blot analysis in several cancer cell lines. The ability of 33B7 conjugated to saporin to inhibit *in vitro* proliferation of PTFRN positive cell lines was investigated, as well as the 33B7 ADC *in vivo* effect on tumor growth in athymic mice. All flow cytometry and *in vitro* internalization assays were analyzed for statistical significance using a Welsh’s T-test. Animal studies were analyzed using Two-Way Analysis of Variance (ANOVA) utilizing post-hoc Bonferroni analysis, and/or Mixed Effects analysis. The 33B7 cell surface target was identified as Prostaglandin F2 Receptor Negative Regulator (PTGFRN), a transmembrane protein in the Tetraspanin family. This target was confirmed by showing that PTGFRN-expressing cells bound and internalized 33B7, compared to PTGFRN negative cells. Cells able to bind 33B7 were PTGFRN-positive by Western blot analysis. *In vitro* treatment PTGFRN-positive cancer cell lines with the 33B7-saporin ADC inhibited their proliferation in a dose-dependent fashion. 33B7 conjugated to saporin was also able to block tumor growth *in vivo* in mouse xenografts when compared to a control ADC. These findings show that screening antibody libraries for internalizing antibodies in cancer cell lines is a good approach to identify new cancer targets for ADC development. These results suggest PTGFRN is a possible therapeutic target via antibody-based approach for certain cancers.

## Introduction

Antibody-Drug Conjugates (ADCs) are a combination of biological and small-molecule drugs that have recently received increased interest as therapeutic choices in oncology. ADCs are composed of a monoclonal antibody (mAb), which specifically binds to a cell surface target, a linker, and a cytotoxic payload. After binding to its cell-surface antigen, the mAb induces endocytosis of said antigen, shuttling the toxic payload inside the cell. Depending on the linker type, the toxic payload is released from the mAb via a cleavable linker, and exits the lysosome either before or during proteolysis [1]. Alternatively, with the use of a non-cleavable linker, the payload cannot enter the cytosol until after the lysosome fully degrades, at which point it is then free to perform its anti-cancer effect [2]. Additionally, there has also been an increased focus on developing ADCs that do not require internalizing into the cell interior for their therapeutic effect. Radioimmunoconjugates are specific antibodies that can bind to cancer cell-surface markers, and deliver localized radiation to the cell due to their conjugation to radioactive isotopes [3]. This effect takes place outside of the cell, with no internalization required. There are also ADCs with non-radioactive conjugates that still rely on extracellular cleavage of their linker, which allows for the diffusion of the cleaved drug across the cell. In fact, the recently approved ADC Troveldy (Sacituzumab govitecan) operates in just such a manner [4]. While several ADCs have been FDA-approved for cancer treatment, the majority of approved ADCs target hematopoietic tumors. The main ADC approved for solid tumor-targeting is Kadcyla, or T-DM1 [5]. T-DM1 is an ADC specifically used to treat HER-2 overexpressing breast cancer. It is derived from the existing anti-HER2 monoclonal antibody Trastuzumab to which emtansine has been conjugated via a non-reducible thioether linker. Once T-DM1 binds and enters the cell, emtansine binds tubulin, leading to cell death by mitotic arrest [6]. In its ADC form, T-DM1 has been reported to be more potent than Trastuzumab. HER-2-positive cancer patients treated with T-DM1 show an improved 3-year disease-free survival rate of 88.3%, compared to 77% in patients treated with Trastuzumab [7]. Only 10.5% of T-DM1 patients suffered distant or metastatic recurrence, compared to 15.9% of patients treated with Trastuzumab, and breast cancer recurrence or death occurred in 12.2% of patients who underwent T-DM1 treatment, as opposed to 22.2% of patients who had Trastuzumab therapy. In addition, patients whose HER-2 expression was immunohistologically scored as lower than 3+ and considered “HER-2 negative” and ineligible for Trastuzumab therapy, were able to benefit from Kadcyla therapeutic effects. These improvements are likely because the monoclonal antibody is capable of binding specifically to HER-2 and blocking its dimerization, interfering with its signaling for increased proliferation, while conjugated emtansine further propagates the anti-cancer effect by triggering apoptosis via mitotic arrest [8]. The clinical benefits obtained with T-DM1 further illustrate a core advantage of ADC therapy compared to treatment with its respective monoclonal antibody alone. They emphasize that coupling the high specificity and activity of the antibody with a potent cytotoxic compound may result in a drug with improved efficacy and enhanced therapeutic window compared to either component separately [9]. Other FDA-approved ADCs include, but are not limited to: Brentuximab Vedotin, which targets CD30/TNFRSF8, Gemtuzumab Ozogamicin which targets CD33/SIGLEC-3, Inotuzumab ozogamicin, which targets CD22, polutuzumab vedotin-piiq, which targets CD79b, Enfortumab vedotin, which targets Nectin-4, and Belantamab mafodotin, which targets CD269 [10–14]. Very recently, the FDA approved the aforementioned Sacituzumab Govitecan, which targets Trop2 for metastatic breast cancer therapy, as well as the next generation in HER2-targeting ADCs, Trastuzumab deruxtecan [15–17]. However, there is still interest in identifying more cancer-related targets that can be the basis for new ADC therapy, particularly for solid tumors with unmet needs for targeted therapies.

Several *de novo* methods to discover internalizing antibodies and their respective targets have been used and reported. The screening of large hybridoma libraries have led to the discovery of several important targets and internalizing monoclonal antibodies [18, 19]. Phage display in *E*. *Coli* has allowed for the rapid generation of large numbers of single chain Fv antibodies that can be used for high-throughput screenings of cancer cell binding [20]. Advancements in the field of artificial intelligence have allowed researchers to work with even larger databases and data sets, allowing for computational technology to more accurately perform tasks such as therapeutic antibody design, epitope prediction, and complementarity-determining region (CDR) optimization, which can also help guide the development of internalizing antibodies [21].

Our approach has centered on the identification of new cell-surface targets that are internalizable, and expressed on cancer cells by the screening mouse hybridoma libraries developed in our laboratory against cancer cell-surface antigens. These antibody libraries are screened for their ability to bind preferentially to the cancer cell surface, internalize, and then deliver a cytotoxic payload to target cells. The selected antibody can then be used to identify its binding protein by proteomics approach. Once identified, the therapeutic potential of the newly discovered cancer cell target can then be investigated. Using this approach, we have characterized an internalizing mouse monoclonal antibody, 33B7, with interesting properties in several cancer cell lines. The present paper describes the properties of this antibody 33B7, identifies its target as Prostaglandin F2 Receptor Negative Regulator (PTGFRN), and shows its efficacy as an ADC in several cancer cell lines.

Identification of PTGFRN by antibody approach has been previously reported by screening human single chain Fv (scFv) phage display antibody library selected for rapid internalization into the SKBR-3 breast cancer cell line [22]. This study also showed that internalization of PTGFRN could be triggered by the binding of a scFv modeled after the antibody Fab region (scFab). The studies presented here also provide the first evidence that internalization of PTGFRN can be coupled with payload delivery in cancer cells, and suggests that PTGFRN could be a novel therapeutic target for further ADC development.

## Materials and methods

### Cell culture

All cell lines were obtained from the American Type Culture Collection (ATCC, Manassas, VA). The hamster spindle cell carcinoma cell line AGSCC-3 was isolated in our laboratory. Unless otherwise specified, all cell cultures were incubated in a 5% CO_2_ incubator at 37°C. HEK-293A cells, as well as all transfected clones derived from HEK-293A cells, were cultured in Dulbecco’s Modified Eagle’s Medium (DMEM; HyClone, Cat. SH30022.02) with 10% fetal bovine serum (FBS). Culture medium for the PTGFRN-transfected HEK-293A cells also contained 600μg/mL of G418. AGSCC-3, A431 (CRL-1555), MDA-231 (CRM-HTB-26), and TOV-21G (CRL-11730) cells were cultured in DMEM/F12 medium (1:1 mixture) supplemented with 50μg/ml Gentamicin and 5% FBS. The human medulloblastoma DAOY (HTB-186) cells were cultured in Eagle’s Minimum Essential Medium (EMEM) with 10%FBS.

### Production of 33B7 mAb

33B7 Hybridoma were cultured in static bioreactor bag in serum-free medium for 14 days. 33B7 antibody was purified from the culture medium by affinity purification through a 10 ml protein-A sepharose using an FPLC NGC machine (Bio-rad) at a flow rate of 5mL/min. The captured antibody was eluted with pH 2.5 Glycine (50mM), and neutralized with pH 8.5 Tris (1M). The antibody was then buffer exchanged by dialysis into PBS pH: 7.4. Antibody purity was checked by SDS-PAGE electrophoresis in reduced and non-reduced conditions.

### Purification of the antibody target protein and identification by mass spectrometry

AGSCC-3 spindle cell carcinoma cells were cultured in 2 Roller Bottles in DMEM/F12 medium supplemented with 5% FBS. After reaching confluency, the cells were detached by incubation with 5mM EDTA in PBS. 5.0x10^7^ cells were then washed twice with 10mL of PBS, and finally re-suspended in 2mL of PBS. 4mg of Thermo Fisher EZ-Link™ Sulfo-NHS-Biotin (Thermo Fisher, Cat. 21217) was added to the cell suspension, and the cells were allowed to mix for 2 hours at 4°C.

After 2 hours, the biotinylation reaction was quenched by spinning down the cells, aspirating the biotin-containing supernatant from the pellet, and washing the pellet 3 times with 10mL of PBS containing 100mM Glycine. Cells were then incubated with a hypotonic solution (10mM HEPES, 15mM MgCl_2,_ 10mM KCl, 0.2mM Sodium Orthovanadate, 0.5mM PMSF) for 20 minutes on ice. After hypotonic shock, cell material was homogenized to promote cell rupture.

Differential centrifugation was then carried out, starting first with a 15-minute centrifugation at 700G in a Sorvall RT6000 Refrigerated Centrifuge to remove the nuclei. The supernatant was then centrifuged at 10,000G for 25 minutes in a Fisher Scientific accuSpin Micro R tabletop centrifuge for organelle removal. Finally, a 60-minute centrifugation at 114,000G in a Beckman J2-21 centrifuge was carried out to isolate the membrane fraction. This pellet was then solubilized in RIPA buffer (EMD Millipore, Cat. 20–188) containing Roche cOmplete^TM^, EDTA-free protease inhibitor cocktail (Millipore Sigma, Cat. 11873580001). After initial solubilization, the solution was sonicated for 20 seconds on ice, with 40 seconds rest on ice. This cycle was repeated three times.

After determining the total protein concentration by MicroBCA, samples containing 1mg each of solubilized membrane protein fraction were immunoprecipitated by incubation with either 40μg of 33B7 antibody, 40μg of non-immune mouse IgG (BioXCell, Cat. BP0083), or 40μg of unrelated control antibody called 21F2 overnight at 4°C. 25μL of protein G agarose beads (Millipore Sigma, Cat. P6649-5ML) were then added to bind the antibody and any antibody-bound proteins. The beads were subsequently centrifuged and washed 5 times with 1mL cold RIPA buffer. Protein G agarose-bound proteins were eluted with pH 1.9 Glycine buffer (50mM Glycine, 25mM NaCl), and neutralized to pH 7.4 with a Tris neutralization buffer (1M Tris, 1.5M NaCl). 25μL of Streptavidin agarose beads (Millipore Sigma, Cat. 16–126) were added to this elution, and incubated for 1.5 hours at 4°C. After washing the beads 5 times with PBS, the beads were treated with 50μL of 2X SDS sample buffer solution containing 200mM DTT (Grainger, Cat. 31FX81) and heated to 95°C for 5 minutes.

To confirm the presence of protein, 10μL of the eluted material was analyzed by SDS-PAGE on two 1mm 4–12% Bis-Tris Bolt gels (Invitrogen, Cat. NP0321BOX). With one of the gels, proteins were transferred onto a PVDF membrane, followed by Western Blot with Streptavidin-HRP to visualize biotinylated proteins pulled down by our antibodies. The second gel was incubated in a colloidal blue staining solution (Thermo Fisher, Cat# LC6025) for 3 hours to stain the proteins. After being de-stained, a prominent 130kDa band was apparent in the gel. This band, and the same area of the gel in the mouse IgG lane, was excised for Mass Spec analysis. In-gel trypsin digestion, followed by HPLC and Mass spectrometry using a Quadrupole-Orbitrap Mass Spectrometer was performed at the Proteomics and Mass Spectrometry Facility of the University of Massachusetts Medical School (Shrewsbury MA). Data was analyzed using Scaffold software.

### Measurement of 33B7 cell surface binding by flow analysis

Cells were collected with PBS-5mM EDTA. 5x10^5^ cells were incubated in 100μL of 5μg/mL mouse IgG or 33B7 mAb in DMEM + 1% FBS for 1-hour at 4°C. Cells were then washed in 200μL cold PBS, and incubated with 10μg/mL Rabbit-anti-Mouse IgG (Jackson ImmunoResearch Laboratories, Cat. 315-005-003) in DMEM + 1%FBS for 1-hour at 4°C. Cells were then washed in 200μL cold PBS, and incubated in 20μg/mL Goat-anti-Rabbit-Alexa 647 in DMEM + 1% FBS for 1-hour at 4°C (Jackson ImmunoResearch Laboratories, Cat. 111-605-045). Cells were washed in 200μL cold PBS, then re-suspended in 160μL PBS, and binding was measured using an Intellicyt Flow Cytometer (Intellicyt HTFC Screening System).

### Examination of 33B7 internalization by immunofluorescent (IF) assay

The protocol used to examine the internalization process with the 33B7 antibody is identical to the Flow analysis assay described above. However, both adherent and suspension cells were used depending on the cell line observed, as well as an additional visualization time point at the end of the experiment.

#### AGSCC-3 IF

Sterile glass coverslips (Thermo Fisher, Cat. 3400) were placed in 35mm dishes (Corning, Cat. 430165), and coated with 33μg/mL Rat Collagen Type 1 (Corning, Cat. 354236) in sterile H_2_O overnight at 4°C. The collagen solution was aspirated the next day, and the dishes and coverslips were air-dried. 1x10^5^ cells were seeded into these dishes and cultured for 2 days. On Day 2, cells were incubated in 1.5mL of 5μg/mL mouse IgG or 33B7 in DMEM + 1% FBS for 1-hour at 4°C. This antibody solution also contained 10μg/mL of DAPI (4′,6-diamidino-2-phenylindole; Thermo Fisher, Cat. D1306) for nuclear counter-staining. Cells were then washed with 2mL cold PBS, and incubated with 10μg/mL rabbit-anti-mouse IgG in DMEM + 1%FBS for 1-hour at 4°C. Cells were washed with 2mL cold PBS, and incubated with 20μg/mL goat-anti-rabbit-rhodamine conjugate (Thermo Fisher, Cat. R-6394) in DMEM + 1% FBS for 1-hour at 4°C. Cells were washed in 2mL cold PBS. For time = 0, the coverslips were immediately removed from the 35mm dishes, and placed on a microscope slide (Thermo Fisher, Cat. 3400), and viewed with an Olympus BX40 fluorescence microscope, through the TRITC filter. The t = 1-hour at 37°C and t = 1-hour at 4°C dishes were placed in their respective incubator/fridge, and incubated for 1-hour, at which point the cells were washed once more with cold PBS. The coverslips were removed, placed on a slide, and viewed in our fluorescent microscope.

#### PTGFRN- and mock-transfected HEK-293A IF

HEK-293A cells were transiently transfected with either human PTGFRN cDNA expression vector, or empty expression vector as described below. After 48 hours transient transfection, cells were collected with PBS-5mM EDTA. 5x10^5^ cells were incubated in 100μL of 5μg/mL mouse IgG or 33B7 in DMEM + 1% FBS for 1-hour at 4°C. This antibody solution also contained 10μg/mL of DAPI (4′,6-diamidino-2-phenylindole) for nuclear counter-staining. Cells were then washed in 200μL cold PBS, and incubated with 10μg/mL rabbit-anti-mouse IgG in DMEM + 1%FBS for 1-hour at 4°C. Cells were then washed in 200μL cold PBS, and incubated in 20μg/mL goat-anti-rabbit-rhodamine conjugate in DMEM + 1% FBS for 1-hour at 4°C. Cells were washed in 200μL cold PBS. At time 0, the cells were then re-suspended in 10μL of PBS, pipetted onto a microscope slide with coverslip, and viewed with an Olympus BX40 fluorescence microscope, through the TRITC filter. In parallel, one aliquot of the cell suspension was placed for 1-hour in a 37°C incubator, and a second aliquot was placed for 1-hour in a 4°C fridge. At the end of the incubation, the cells were washed with cold PBS, re-suspended in 10μL of PBS, pipetted onto a microscope slide with coverslip, and viewed with an Olympus BX40 fluorescence microscope, through the TRITC filter. All images were taken with TSView software (Version 7), and composite images were generated using ImageJ software

### PTGFRN cDNA transfection into HEK-293A cells

Full-length human PTGFRN cDNA was synthesized into pcDNA3.1 (+) vector (Invitrogen, Cat. V79020). The PTGFRN cDNA expression vector plasmid was transiently transfected into HEK-293A cells with a Lipofectamine transfection kit (Thermo Fisher, Cat. 11668027). 4x10^5^ HEK-293A cells were plated in a 6-well plate. 24 hours later, 3 wells were transfected with an empty cDNA pcDNA 3.1 vector as a mock transfection negative control, while the other 3 wells received the PTGFRN plasmid. After 5 hours incubation with Lipofectamine and plasmid DNA, the mixture was aspirated from the cells, and replaced with complete serum-supplemented medium. For transient transfection, the cells were collected after 48 hours, with one well used to collect lysate (100μL RIPA lysis buffer with protease inhibitor cocktail), while the remaining cells were harvested with 5mM EDTA, and Flow Cytometry and Immunofluorescence were performed as described above.

### Inhibition of proliferation with 33B7 and Fab-ZAP

In a 96-well plate, 2000 cells/well were plated in triplicate in 100μL of DMEM/F12 medium supplemented with 2.5% FBS, 0.4ug/ml 33B7 antibody, and 0.9ug/ml of anti-mouse Fab-conjugated with Saporin (Fab-ZAP, Advanced Targeting Systems, Cat. IT-48). As an isotype control, cells were incubated with mouse IgG (instead of 33B7) and Fab-ZAP. Cells incubated with 33B7 and mouse IgG without Fab-ZAP were also used as experimental negative controls. After 72 hours, cell proliferation was measured by CellTiter-Glo® Luminescent Cell Viability Assay reagent (Promega, Cat. G7572) according to manufacturer’s instructions. The luminescence levels were measured with a SpectraMax M2 (Molecular Devices, San Jose, CA).

After testing the antibody with the secondary antibody-saporin conjugation, we then tested our 33B7 directly conjugated to saporin in the same assay at various concentrations up to 10nM.

### Direct conjugation of antibody

Purified 33B7 antibody was sent to Advanced Targeting Systems (San Diego, CA), where saporin was directly conjugated to the Fc region of 33B7 using their proprietary cleavable linker.

Biological activity of the directly conjugated 33B7-saporin was determined by the in vitro proliferation assay described above.

### *In vivo* mouse xenograft studies

These studies were carried out in strict accordance with the recommendations of the Guide for the Care and Use of Laboratory Animals from the National Institutes of Health. The protocol used for these studies was approved by the Institutional Animal Care and Use Committee (IACUC) of A&G Pharmaceutical (Protocol number: GS-01). All animal procedures were performed under isoflurane anesthesia and all efforts were made to minimize animal suffering.

Animals were housed in the Institution’s AAALAC-accredited and OLAW certified animal facility, (OLAW D16-00700). Experiments were carried out following A&G’s IACUC-approved procedures. Animal rooms were humidity and temperature controlled with a 12-hour light/dark cycle. 6 to 8-week-old female Athymic nude mice (Charles River Laboratories, Wilmington, MA) were housed with no more than 5 mice/cage aseptically in autoclaved plastic cages with filter top containing autoclaved 1/8” corncob bedding, 18% protein, 9% fat, extruded rodent pellets, and sterile tap water. Cages, food, and water were replaced once a week, using aseptic techniques.

At the time of the experiment, 30 mice were subcutaneously injected with cells in 1:1 volume of Matrigel (Corning, Cat. 354248) of either A431 cells (5x10^5^ cells/mouse) or AGSCC 3 cells (5x10^6^ cells/mouse). The mice were monitored until the subcutaneous tumor reached approximately 80-100mm^3^. After measuring tumor size as described below, mice were randomized into respective experimental groups and ear tagged for the experiment. Animals with tumors that were too large or too small to fit into randomization were not used. The number of mice per group was determined by power calculation based on the hypothesis of an at least 50% difference in tumor volume between the test and control groups.

For A431 cells, 15 mice were then randomized into three experimental groups of 5 mice each: 33B7 antibody conjugated to saporin (33B7-ADC), antibody control 21F2 conjugated to saporin (21F2-ADC) and vehicle control with 5 animals per group. For AGSCC-3 cells, 12 mice were randomized into two experimental groups (6 mice per group with 3 mice per cage) with 33B7-ADC and the antibody control group consisting of mouse IgG-saporin conjugate (Advanced Targeting Systems). For A431 cells, the treatment consisted of once-a-week tail vein injection of 33B7-saporin ADC (10μg/mouse) with two control groups consisting of mice injected with equal volume of either PBS or control ADC 21F2-saporin ADC (10 μg/mouse). For AGSCC-3 xenografts studies, the treatment was performed as described above except that the antibody control group consisted of non-immune mouse IgG-saporin ADC instead of 21F2-ADC.

Mice were monitored daily for general health, sign of distress, and/or tumor necrosis. If a mouse within a cage showed any signs of illness, the mouse was isolated in a new cage with enrichment objects and monitored for any change in health. Humane endpoints were incorporated into animal procedures. Mice were euthanized once tumor volume exceed 3000mm^3^, whenever tumor ulceration/necrosis became severe, or if mice were persistently ill, even after isolation and monitoring. For the AGSCC-3 Xenograft, our control mice had a mortality rate of 30%, while our 33B7-Saporin ADC-treated mice had a mortality rate of 10%. These tumors proliferated extremely rapidly, likely explaining why more control mice died at the later portion of their experiments. For the A431 xenograft experiments, our control mice had a mortality rate of 10%, while our 33B7-Saporin ADC-treated mice had a mortality rate of 0%. Any tumor ulcerations typically coincided with other humane endpoints, or occurred at the end of the experiment, so termination of the mice was prompt in order to reduce potential pain and distress.

Tumor measurements were taken twice a week aseptically with a caliper, and volume was calculated using the formula (L x (W^2^)/4). For each mouse, tumor growth rate was measured as the tumor volume on day of measurement divided by the initial tumor volume on Day 1. At the end of the experiments, the mice were euthanized following approved guidelines by isoflurane anesthesia, exsanguination by cardiac puncture, followed by cervical dislocation. Both experiments were repeated 3 times.

### Statistical analysis

Statistical analyses were carried out using GraphPad Prism version 8.3. All flow cytometry and *in vitro* internalization and proliferation assays were carried out in triplicates and repeated three times. Results were analyzed for statistical significance using a Welsh’s T-test.

Animal studies were analyzed at each time point between the groups using Two-Way Analysis of Variance (ANOVA) utilizing post-hoc Bonferroni analysis, and/or Mixed Effects analysis wherever necessary. For all statistical analysis, significance was determined at P < 0.05 (*).

## Results

### Binding and internalization of 33B7 monoclonal antibody to cancer cells

**Fig 1:**
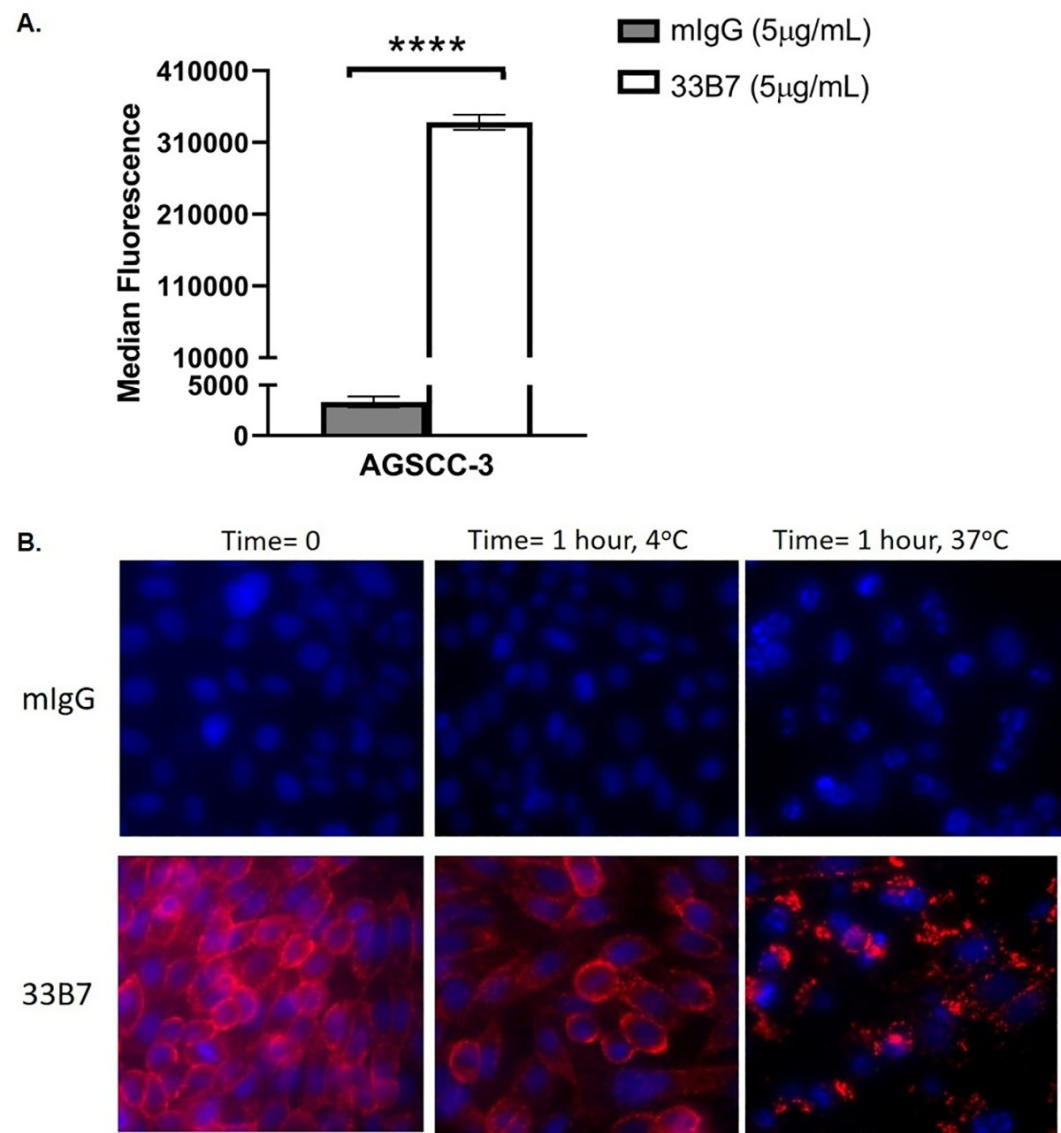
33B7 binding and internalization in cancer cells. (A) The spindle cell carcinoma cell line AGSCC-3 was tested for 33B7 binding by Flow Cytometry and showed high levels of binding. (B) AGSCC-3 cells show immunofluorescence binding by 33B7 (red), and not mouse IgG. Cells allowed to incubate at 37C for an hour show a punctate appearance, signifying internalization of our fluorescent-labelled antibody. Cells incubated at 4C do not show this internalization. DAPI (blue) was used to counterstain nuclei.

For target discovery, our laboratory has developed mouse hybridoma libraries by using plasma membrane-enriched fractions as source of antigens for mice immunization. Hybridoma supernatants were then tested by flow assay for binding to a variety of cell lines. Antibodies secreted from selected hybridomas were then purified and further characterized. In this study, we selected and examined one such antibody called 33B7. Flow binding assay to examine 33B7 binding on cell surface identified the spindle cell carcinoma cell line AGSCC-3 as highly positive for binding (Fig 1A). In addition to the binding to the cell surface of AGSCC-3 cells, 33B7 was internalized when cells were placed at 37°C for 1 hour, as shown in Fig 1B.

### Purification of 33B7 cell surface target

AGSCC-3 cells, which showed a high level of 33B7 binding by flow assay (Fig 1A), was used for purification and identification of the 33B7 cell surface target.

**Fig 2:**
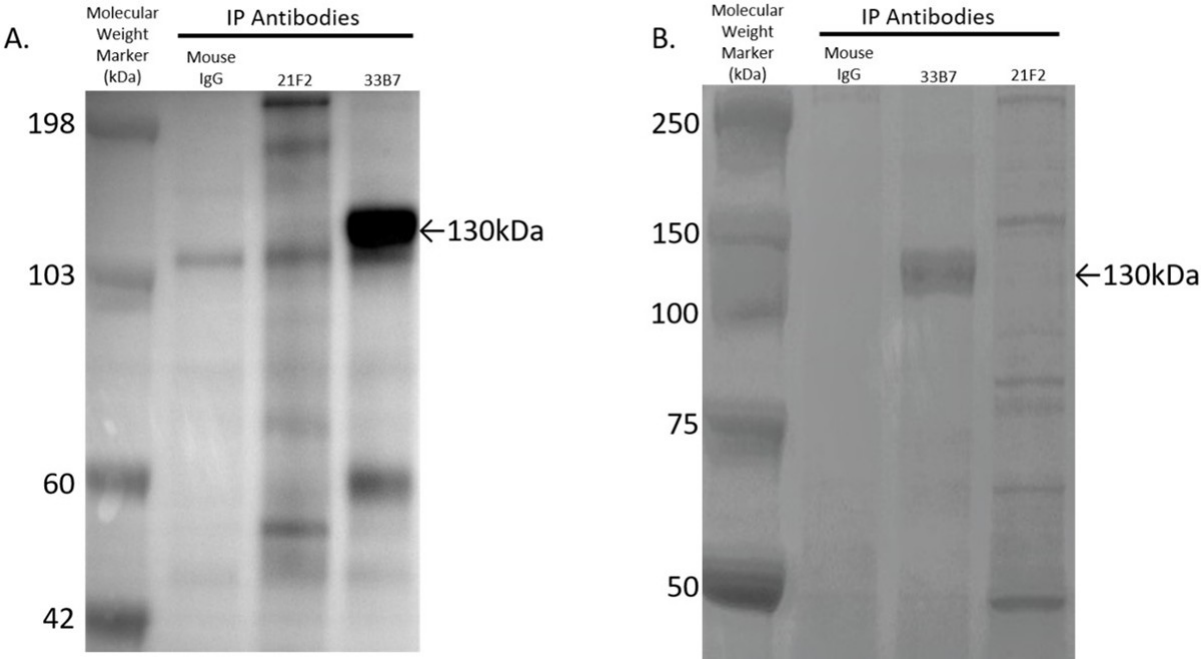
Immunoprecipitation with 33B7 pulls down a unique 130kDa protein. 33B7 and two negative control antibodies were used to immunoprecipitate (IP) the biotinylated membrane fraction of AGSSC-3 cells. (A) Western Blot using Streptavidin-HRP on IP elutions of biotinylated membrane fraction. (B) Coomassie gel staining of IP elutions. A 130kDa band was visible in both assays, and only seen in the fraction immunoprecipitated with the 33B7 mAb.

Details of the purification procedures are provided in the Material and Method section. Briefly, the cell-surface proteins were biotinylated, followed by plasma membrane preparation, solubilization, and immunoprecipitation by incubation with 33B7 antibody, or with unrelated antibodies used as negative controls, followed by protein G beads capture as described in the method section. Proteins bound to the 33B7-protein G beads or control antibody-protein G beads were eluted by acidic pH and the resulting supernatants were neutralized and incubated with streptavidin beads to bind the biotinylated proteins eluted from the antibodies protein G beads. After incubation, beads were washed, mixed with SDS-sample buffer and the bound proteins were resolved by SDS-polyacrylamide gel electrophoresis on a 4–12% Bis-Tris polyacrylamide gel. The gel was then transferred to a PVDF membrane for western blot analysis to detect biotinylated proteins with Streptavidin-HRP incubation as described in the method section. As shown in Fig 2A, a major protein band with an apparent molecular weight of 130kDa was detected by HRP-streptavidin western blot analysis. At this point, the remaining IP elution was run on an additional gel, and this time was stained with Coomassie Blue stain. A major band with a similar apparent molecular weight was also detected on the gel stained with Coomassie Blue (Fig 2B). This band was not present in samples from the same cell extract that had been immunoprecipitated with non-immune mouse IgG, or an unrelated antibody control 21F2.

The gel fragments corresponding to the position of the 130kDa band detected by Coomassie Blue were excised from the 33B7 lane and from the mIgG lane and sent to the Proteomics and Mass Spectrometry Facility of the University of Massachusetts Medical School for in-gel trypsin digestion, HPLC, and Mass Spectrometric analysis.

### Mass spectrometric identification of the cell surface target

After analyzing the data received from the Mass Spec Facility, the protein with the highest number of identified peptides in the 33B7 lane was identified as Prostaglandin F2 Receptor Negative Regulator, or PTGFRN (ID number Q9P2B2). Table 1 shows the top 5 most abundant proteins identified. We can see that the band excised from the 33B7 IP lane contained 246 PTGFRN-unique peptides, compared to just 53 in the corresponding excised mIgG portion, with PTGFRN being by far the most abundant protein identified.

**Table 1:** Mass spectrometric analysis of 33B7 immunoprecipitation elution.

Protein Name	Accession Number	Molecular Weight (kDa)	33B7 Unique Peptide Number	Mouse IgG Unique Peptide Number
Prostaglandin F2 Receptor Negative Regulator	Q9P2B2	94	246	53
Keratin, Type II cytoskeletal	P04264	94	80	41
Desmoplakin	P15924	328	39	5
Vimentin	P08670	54	15	16
Junction Plakoglobin	P14923	128	15	4

This table shows the 5 most abundant proteins detected in the mass spectrometric analysis of the 33B7 IP. PTGFRN is the most abundant protein immunoprecipitated by 33B7 and is most likely to be the 33B7 antigen.

### HEK-293A cells transiently transfected with PTGFRN cDNA can bind 33B7 antibody

**Fig 3:**
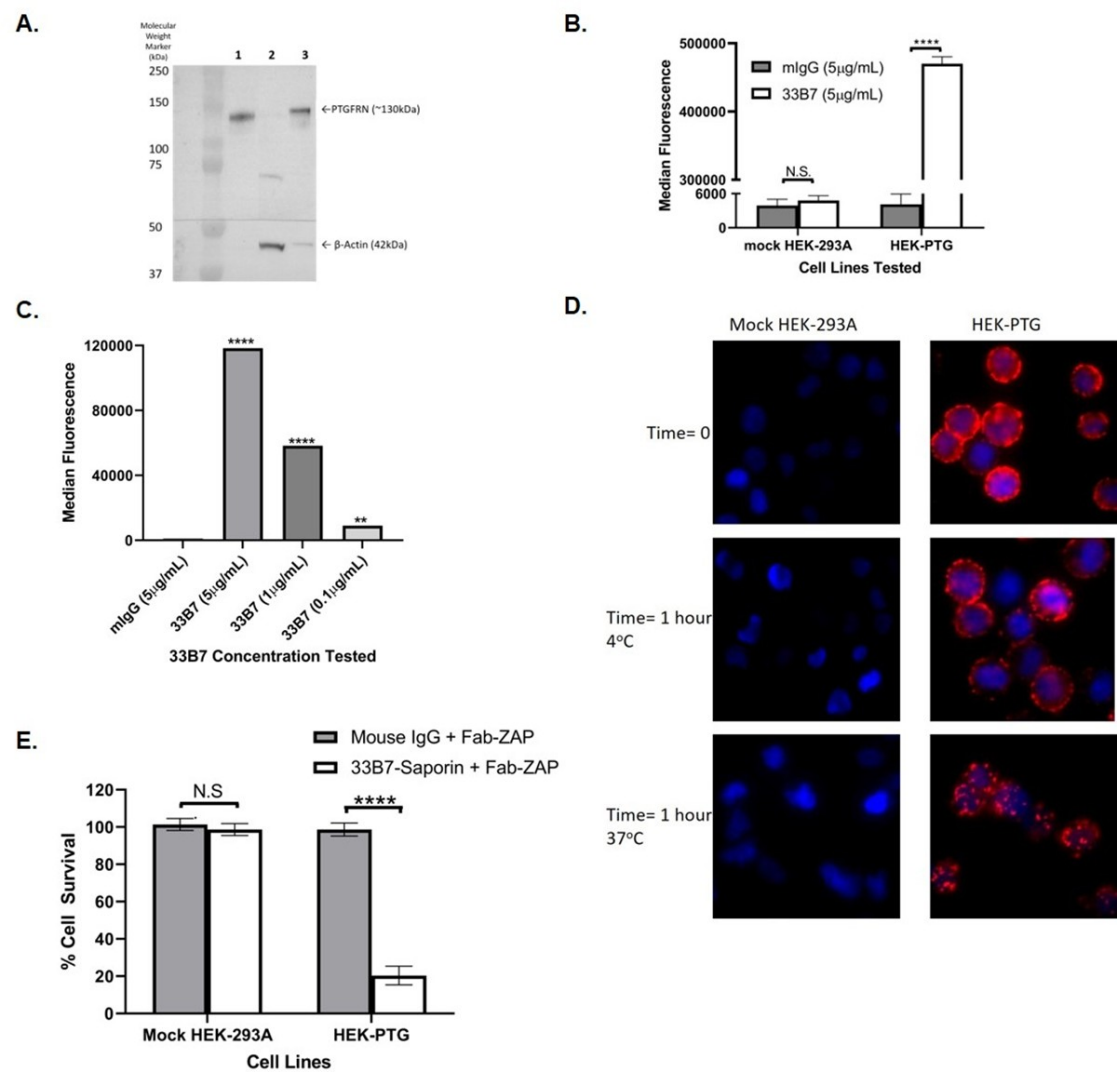
Visualization of 33B7 binding and internalization with payload delivery in mock- and PTGFRN-transfected HEK-293A cells. (A) PTGFRN expression visualized by Western Blot in (1) AGSCC-3 (5μg), (2) HEK-293A (40μg), and (3) HEK-PTG (5μg). **Note:** Actin was not visible in the AGSCC-3 cell line due to lower protein amount being sufficient to detect PTFRN. (B) HEK-293A cells were transiently transfected with either empty pcDNA3.1 expression vector (mock), or PTGFRN cDNA expression vector (HEK-PTG) as described in the methods section. We then measured 33B7 binding to both cell lines compared to mIgG by Flow Cytometry. (C) HEK-293A cells transiently transfected with PTGFRN cDNA expression vector (HEK-PTG) as described in the methods section were analyzed via FACS, and the 33B7 antibody concentration was titered down to establish a dose response. (D) Mock HEK-293A cells and HEK-PTG cells were used to visualize 33B7 binding and internalization by immunofluorescence as described in the method section. DAPI was used to counterstain the cell nuclei (Blue). (E) Mock HEK-293A and HEK-PTG cells were incubated with 33B7 (1nM) and the anti-mouse Fab-ZAP (9nM). After 3 days, ATP levels were measured using the CellGlo Assay. Statistical analysis was shown via a Welch’s t-test between control (Mouse IgG-Saporin) and 33B7-Saporin (****, P < 0.0001).

Experiments were carried out to verify that PTGFRN identified by mass spectrometry was the target protein binding to the 33B7 antibody. For this purpose, as described in the methods section, we inserted human PTGFRN cDNA into a pcDNA3.1 vector, and transiently transfected it into HEK-293A cells, which are negative for PTGFRN, as shown by western blot analysis (Fig 3A). The resulting PTGFRN expressing cells were used to examine 33B7 binding by flow cytometry, comparing mock transfected HEK-293A cells vs. PTGFRN transfected cells (HEK-PTG). As shown in Fig 3B, the HEK-PTG cells showed a large increase in binding by 33B7 while mock transfected HEK-293A cells showed no 33B7 binding, confirming the mass spectrometry results identifying PTGFRN as the 33B7 binding protein. Fig 3C shows that this 33B7 binding to PTGFRN is dose-dependent.

### 33B7 induces internalization of PTGFRN

Since 33B7 was binding on the cell surface of PTGFRN-transfected (HEK-PTG) cells, we then tested by immunofluorescence whether the transfected PTGFRN protein was also internalized by 33B7. After antibody binding at 4C, the cells were placed in a 37C incubator for 1-hour followed by observation under a fluorescent microscope. Fig 3D shows strong cell surface binding of 33B7 on the PTGFRN-transfected HEK-PTG cells maintained at 4C, with no visible binding on the mock-transfected HEK-293A cells. After 1-hour incubation at 37C, the immunofluorescence (red) was intracellular and had a punctate appearance, indicating internalization of PTGFRN had occurred at 37C upon binding of the 33B7 antibody. The 33B7 binding remained on the cell surface for the cells maintained at 4C for 1 hour, instead of 37C indicating that no internalization had occurred at 4C.

### Inhibition of proliferation of PTGFRN-transfected HEK-293A cells, as well as natural cancer cells, by internalization of 33B7

After confirming that 33B7 could bind and internalize cell-surface PTGFRN on the HEK-PTG cells, we next examined whether it could be used to deliver a toxic payload to cells expressing this target. As a proof-of-concept, we chose to use a saporin-conjugated Fab fragment anti-mouse IgG antibody, which is commercially available, and is widely used for internalization studies [23] Saporin is a 30kDa Ribosome-inactivating protein purified from the seeds of the Soapwort plant (*Saponaria officinalis)*. It acts as an N-glycosidase and cleaves RNA that codes for the 28S subunit of the ribosome. This results in inactive ribosomes, and thus, cell death. However, saporin is unable to pass through the cell membrane by itself [24]. The only way it can reach the ribosomal RNA is by conjugation to an internalizing antibody, or other agent able to enter the cell. This makes it a great tool to demonstrate proof of concept for payload delivery via 33B7 antibody. Treatment with the internalizing anti-PTGFRN 33B7 antibody either directly conjugated to saporin, or in combination with an anti-mouse secondary antibody conjugated to saporin (Fab-ZAP), should result in inhibition of cell proliferation of 33B7-positive cells.

As shown in Fig 3E, HEK-PTG proliferation was inhibited by 80% ± 5% by treatment with 33B7 and Fab-ZAP, as described in the methods section. We also tested the sensitivity of the cell lines under investigation to unconjugated saporin alone and found that the lowest concentration that we saw toxicity was 1μM (data not shown). This concentration greatly exceeds the working concentration of saporin that would be used with our conjugates, which is approximately 25nM for our *in vitro* assays, and approximately 100nM in our *in vivo* assays.

At this point, we had the 33B7 antibody custom conjugated directly to saporin, resulting in our proof-of-concept ADC. Mouse IgG conjugated with saporin was used as a negative isotype control.

**Fig 4:**
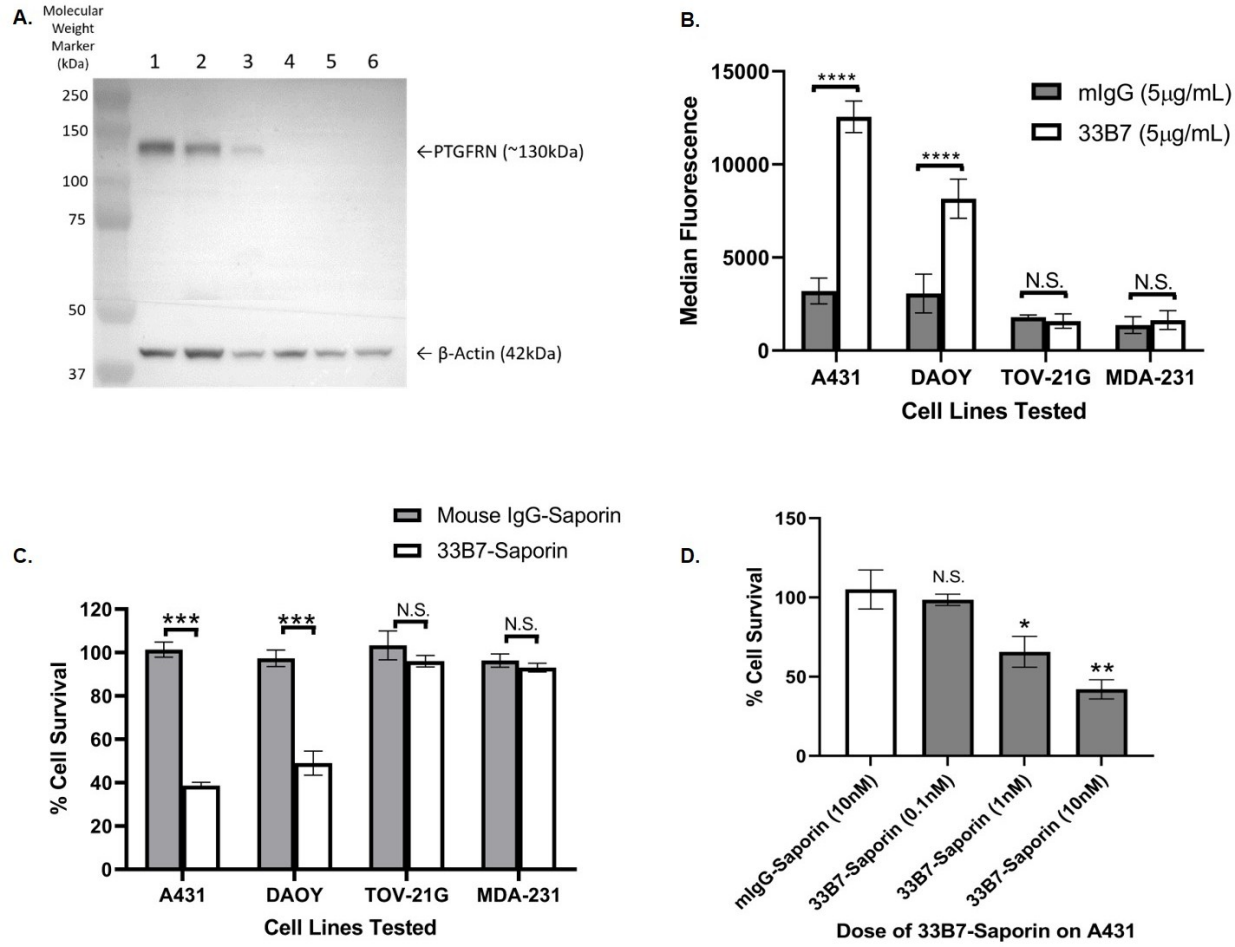
PTGFRN expression and effect of 33B7 on various cancer lines. (A) PTGFRN expression by Western Blot with anti-PTGFRN antibody in 1. A431 (40μg), 2. DAOY (40μg), 3. MSTO-211H (40μg), 4. JEG-3 (40μg), 5. TOV-21G (40μg), and 6. MDA-231 (40μg) cell lysates. (B) Several cancer cells were screened for 33B7 cell surface binding by flow assay. A431, DAOY showed 33B7 binding compared to a mouse IgG control while TOV-21G and MDA-MB-231 showed no 33B7 binding. (C) Dose response with 33B7-Saporin ADC on A431 cells over 3 days shows decrease in proliferation compared to cells treated with Mouse IgG-Saporin conjugate (10nM). (D) Effect of 33B7-ADC on various cancer cell lines. Treatment with 33B7-Saporin conjugate (10nM) over 3 days shows decrease in proliferation compared Mouse IgG-Saporin conjugate (10nM). Cancer cells with undetectable PTGFRN expression levels (TOV-21G, MDA-231) showed no growth inhibitory response. Statistical analysis was shown via a Welch’s t-test between control (Mouse IgG-Saporin) and 33B7-Saporin (**, P < 0.01; ***, P < 0.001; ****, P < 0.0001).

Western blot analysis using an anti-PTGFRN antibody was used to examine PTGFRN expression in several cancer cell lines (Fig 4A). Among the human cell lines investigated, A431, DAOY, and MSTO-211H cells were positive for PTGFRN, with the highest expression being in A431 cells. JEG-3, TOV-21G, and MDA-231 cells were found to be PTGFRN negative. We then looked at levels of 33B7 binding to these cells by flow cytometry (Fig 4B). 33B7 binding occurred in a proportional manner. These cell lines were then used to examine the effect of the 33B7-ADC on *in* vitro cell proliferation. Fig 4C shows that, compared to the saporin IgG negative control, PTGFRN-positive human cancer cells treated with the 33B7-ADC resulted in a 61% ± 2% reduction of proliferation in A431 cells, and a 51% ± 6% reduction on proliferation in DAOY cells. No growth inhibitory effect of the 33B7-ADC was observed for the PTGFRN-negative cell lines (TOV-21G, MDA-MB-231). Fig 4D further indicates a dose-dependent inhibition of A431 cell proliferation.

### *In vivo* growth inhibitory effect of saporin-toxin conjugated 33B7

Since the AGSCC3 and A431 cell lines showed the highest level of PTGFRN expression, as well as the highest response to 33B7-ADC, these cell lines were selected to examine the effect of 33B7-ADC on *in vivo* tumor growth in mouse xenograft model when compared to isotype control ADC.

**Fig 5:**
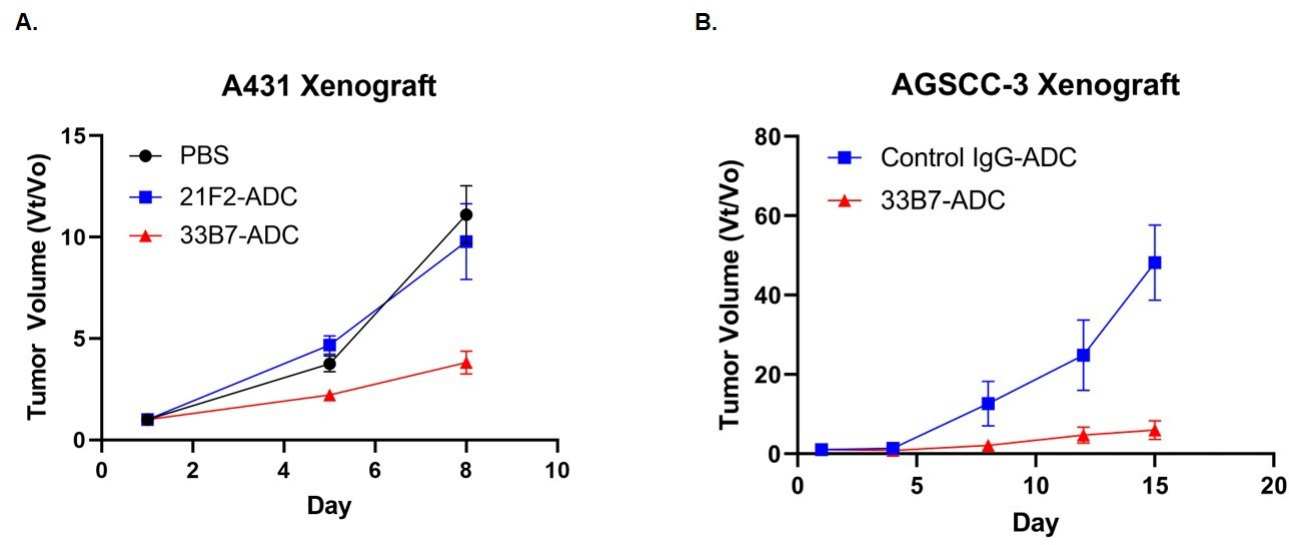
Effect of 33B7-saporin on tumor growth of A431 cells and ASGCC3 cells in athymic nude mice. *In vivo* effect of treatment with 33B7-Saporin in athymic nude mice xenografted with (A) A431 cells, and (B) AGSCC-3 cells. Isotype control (Blue Square; 10μg/mouse of saporin-conjugated isotype control) or 33B7-Saporin (Red Triangle;10μg/mouse) were administered once weekly via tail vein injection. PBS (Black Circle) volume equivalent was used as vehicle control. Tumor sizes were measured biweekly with calipers. Statistical analysis showed significance (at least *, P < 0.05) between the Isotype/Vehicle control and 33B7-Saporin for each time point after start of treatment.

As shown in Fig 5, the treatment of A431 cells (Fig 5A) with weekly intravenous (i.v.) administration of 33B7-saporin ADC (10μg/mouse) resulted in a reduction of tumor growth by at least 50% when compared to mice treated with vehicle control or with isotype control 21F2-ADC. 80% Tumor inhibitory effect of 33B7-ADC was also observed with ASGCC3 cells (Fig 5B) when compared to IgG-saporin antibody control.

## Discussion

Using the selected mouse monoclonal antibody 33B7 to carry out immunoprecipitation, followed by mass spectrometry analysis, our laboratory has identified Prostaglandin F2 Receptor Negative Regulator (PTGFRN) as an internalizable cell-surface target expressed on several cancer cells. The identification of PTGFRN carried out with spindle cell carcinoma cells was confirmed in human head and neck cancer A431 and medulloblastoma DAOY cells by immunoprecipitation with the 33B7 antibody followed by mass spectrometry analysis (Data not shown). The validation of PTGFRN as the target for 33B7 antibody was performed by showing that HEK-293A cells, which cannot bind the 33B7 antibody, and do not express PTGFRN, were able to bind 33B7 when the cells were transfected with human PTGFRN cDNA, as shown by flow binding assay and by immunofluorescence staining. 33B7 was also able to internalize PTGFRN as shown by immunofluorescence, as well as inhibit cell proliferation of PTGFRN-expressing cell lines by toxic payload delivery, either directly via a 33B7-ADC, or indirectly via an anti-mouse secondary antibody saporin conjugate. We have demonstrated a statistically significant and dose-dependent efficacy of the 33B7 ADC in inhibiting the proliferation of several cancer cell lines that express PTGFRN, when compared to control ADC antibody. Using A431 cells and AGSCC-3 cells as mouse xenografts models, we show that 33B7-ADC inhibited tumor growth when compared to control-ADC antibody. Further in vivo studies are required to explore different doses of the ADC, as well as investigating the effect of 33B7-ADC on mouse xenografts models of medulloblastoma and mesothelioma, which we have shown to express PTGFRN, in order to investigate the efficacy of targeting PTGFRN for these cancers with unmet needs.

Additionally, previous studies in optimizing payload delivery to cellular interiors by ADC have identified multiple factors that determine the efficacy of ADC internalization. Receptor recycling of the target protein can re-release an ADC back to the cell exterior, and the presence of the neonatal Fc receptor (FcRn) can also shuttle the ADC back to the cell surface, both of which lower the payload concentration inside the cell. Importantly, rate of internalization can differ between antibodies based on their biochemical properties, so analysis of the binding affinity and rate of internalization should be considered when developing or selecting prospective antibodies. Once an ADC actually gets inside the cell, and begins to be trafficked to the lysosome, the chemistry of a given linker may also affect how well the payload dissociates from the antibody, and how much can accumulate inside the cell [25]. Cleavable linkers typically result in slightly higher amounts of free payload inside the cell, and these linkers can be designed to cleave at various acidic pH levels. Doing so can allow one to control at which point a payload is cleaved after internalization, whether this will occur in the early endosome, late endosome, or in the lysosome itself [26]. All these factors should be considered when trying to develop an ADC, and will be taken into consideration in further optimization of our anti-PTGFRN antibodies.

The PTGFRN protein is a member of the subfamily of proteins known as tetraspanins, characterized by four transmembrane domains that play a role in many aspects of cell biology and physiology. PTGFRN has been shown to affect a wide range of biological processes such as fertilization [27, 28], migration [29], and accumulation of lipids in preadipocytes [30], extracellular vesicle bioactivity [31], non-alcohol fatty liver disease [32], and Alzheimer’s disease [33]. While additional investigation needs to be done to elucidate the exact role the PTGFRN protein plays in these processes, the PTGFRN gene itself has also been identified as a possible predictor for risperidone activity in patients with schizophrenia [34].

Tetraspanins are proteins which bind and interact with multiple partners, some of which can be other tetraspanin proteins forming what is knowns the “tetraspanin web”. For example, PTGFRN is known to associate with several other tetraspanin proteins such as CD81 and CD9 and CD151 [35]. This “web” and its members also serve as signaling molecules for a wide range of biological processes. While the molecular mechanism of these interactions are not well studied, it has been hypothesized that tetraspanins binding to each other may facilitate and strengthen the binding of the tetraspanins to other partners, such as PTGFRN associating with CD151, but only after complexing itself with CD9 [36]. The binding of CD9 to the metalloprotein CD10 also improves the latter’s release via exosomes [37].

Tetraspanins are the basis of complexes known as Tetraspanin-Enriched Microdomains (TEMs). These complexes are made up of many different tetraspanins, bound to a multitude of cytosolic and transmembrane proteins, which can rearrange depending on biological stimuli. Because of this ability to efficiently reorganize themselves, TEMs have been found to facilitate signaling in many different cellular pathways by acting as scaffolds for protein interaction and/or stabilization, such as inhibiting diffusion of integrin α6 through the cell membrane so it is available for migration, but also being able to bind and stabilize integrin α3β1 [38, 39]. Unfortunately, in-depth research on PTGFRN as a cancer therapy target specifically is quite lacking. However, our research on PTGFRN should serve as a first step in further characterizing PTGFRN, as well as exploring its potential role in malignancy.

As mentioned earlier, PTGFRN has been previously identified as binding to one of 13 antibodies selected by human single chain Fv phage display antibody library screening for rapid internalization in SKBR3 cells [22]. This study also showed that internalization of PTGFRN could be triggered by the binding of a single chain fusion variant (scFv) and that co-expression of the scFv and PTGFRN in cells could trap PTGFRN in the ER, without affecting expression of PTGFRN’s binding partners, CD9 and CD81 [22]. Previous research has shown that at the mRNA level, PTGFRN expression is increased in metastatic cancer cells [40]. An interesting question that needs further exploration is the role of PTGFRN in cancer cells and whether its expression is correlated with aggressiveness.

Moreover, PTGFRN has been found to be associated with biological functions that play important roles in tumor development. Aguila et al. showed that PTGFRN was overexpressed in glioblastoma, and higher expression of PTGFRN in tumors correlated with worse survival. These authors also determined that inhibition of PTGFRN by shRNA also increased the radiosensitivity of glioblastoma tumors, as well as decreased cell proliferation and tumor growth [41]. In a paper published by Colin et. al, PTGFRN was found to be essential for angiogenesis, a necessary process in tumor growth. They also showed that treatment of tumor-bearing mice with a truncated mutant form of PTGFRN acting as a dominant negative regulator resulted in inhibition of *in vivo* angiogenesis and tumor growth [42].

Our work here is the first to connect the upregulation and internalization of PTGFRN in various cancer cells with the ability to target PTGFRN via monoclonal antibody to inhibit *in vitro* proliferation and *in vivo* tumor growth, thus paving the way for the development of PTGFRN antibody-drug conjugate for these cancer types. Due to the fact that PTGFRN is found to be further upregulated in metastatic cancers, an ADC would be more attractive as a potential targeted therapy. Finally, we have outlined our hybridoma library screening process in this paper, thus making it possible to apply to other cancer cell types, which may help to identify additional potential targets for ADC generation. By approaching target discovery in such a manner, more tailored therapies could become viable options in target discovery, as well as targeted therapy development in oncology. These results are encouraging to explore the role and effect of ADC targeting PTGFRN for cancers such as mesothelioma and pediatric medulloblastoma, which we have found to both express PTGFRN, and have limited therapeutic options. These studies are on-going.

## Conclusions

In summary, PTGFRN is a cell-surface protein that is upregulated in certain cancer types, including head and neck (A431) and, notably, pediatric medulloblastoma (DAOY), an aggressive cancer with limited therapeutic options. With the selection of the mouse monoclonal antibody 33B7, we have identified PTGFRN as a potential therapy target, and shown that it is internalized by incubation with 33B7. As proof-of-concept, we show that an antibody-drug conjugate (ADC) of 33B7 conjugated with saporin was effective in inhibiting *in* vitro cell proliferation and *in vivo* tumor growth of PTGFRN-positive cancer cells A431 and AGSCC-3 cells. These findings suggest PTGFRN as a novel target for future Antibody-Drug Conjugate development, particularly for pediatric medulloblastoma.

## Supporting information

S1 Raw images Original blot and gel images. Raw, unedited images of gel stains and western blots.
(PDF)
