# Supplementary material for: Identification of Prostaglandin F2 Receptor Negative Regulator (PTGFRN) as an internalizable target in cancer cells for antibody-drug conjugate development
Source: PLoS One. 2021 Jan 27;16(1):e0246197. doi: 10.1371/journal.pone.0246197 (PMC7840024; doi:10.1371/journal.pone.0246197)
Supplement: S1 Raw images — Raw, unedited images of gel stains and western blots. (PDF) [file pone.0246197.s001.pdf]

Marker 1 2 3

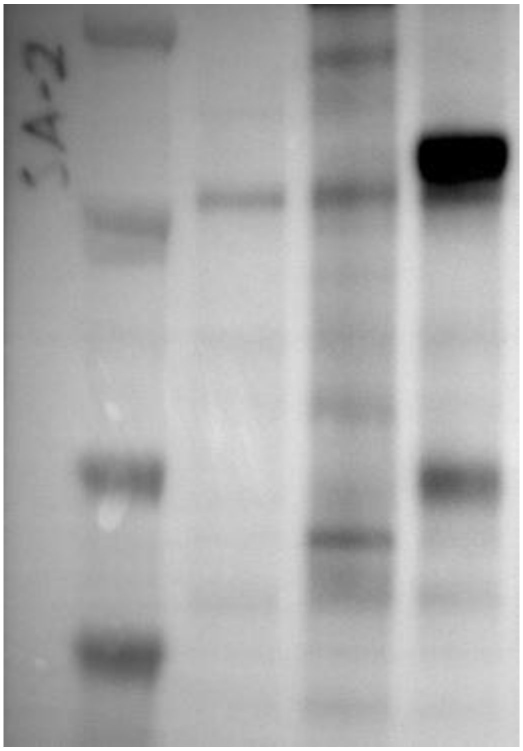

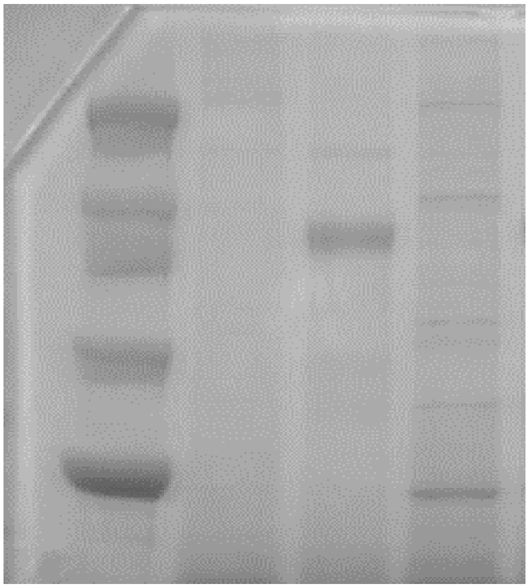

Marker 1 2 3

Blot Information

This blot was used for Fig 2A and 2B, respectively. The Top blot was chosen to show Streptavidin-HRP detection of biotinylated proteins Immunoprecipitated by our chosen antibodies

Top Blot

1= AGSCC-3 Immunoprecipitated by non-immune mouse IgG

2= AGSCC-3 Immunoprecipitated by isotype control mAb 21F2

3= AGSCC-3 Immunoprecipitated by candidate mAb 33B7

Bottom Gel

1= AGSCC-3 Immunoprecipitated by non-immune mouse IgG

2= AGSCC-3 Immunoprecipitated by candidate mAb 33B7

3= AGSCC-3 Immunoprecipitated by isotype control mAb 21F2

Images were captured using Chemiluminescent solution and UVP machine

Marker 1 X X 2 3

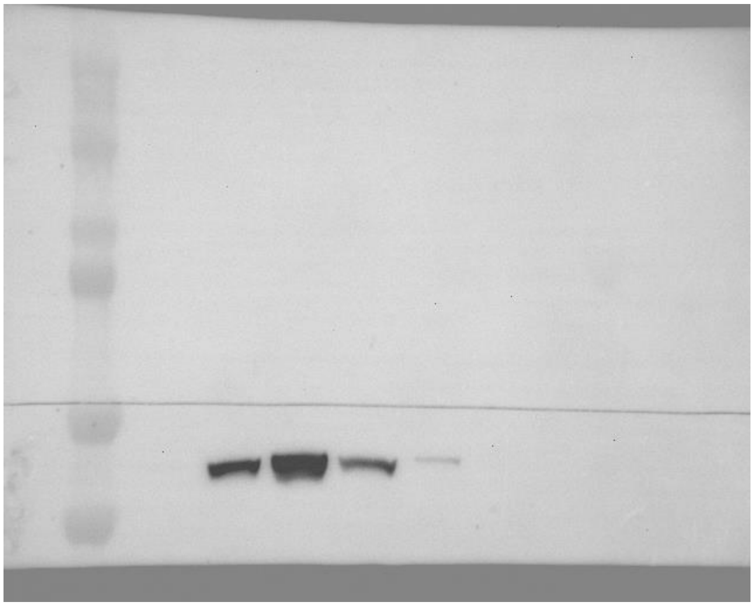

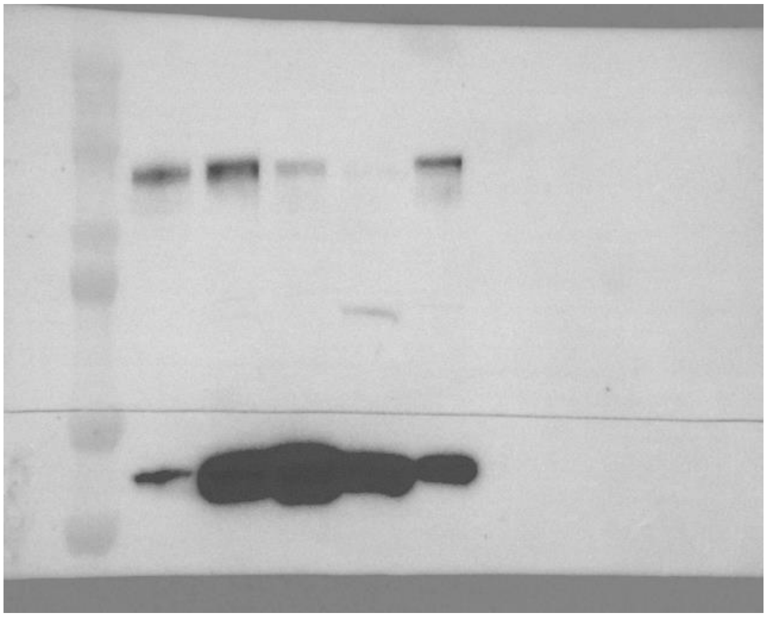

Marker 1 X X 2 3

Blot Information

This blot was used for Fig 3A. The Top blot was chosen to show actin loading, and the bottom blot was used to show the PTGFRN expression. Two lanes were edited out to reduce redundancy in Figure 4.

It should be noted that this membrane was cut in order to incubate the two sections in different primary WB antibodies, with the upper half of the membrane being probed with anti-PTGFRN, and the bottom half of the membrane was probed for anti-Actin.

1= AGSCC-3 5µg

2= HEK-293A 40µg

3= HEK-PTG 5µg

Images were captured using Chemiluminescent solution and UVP machine

Marker 1 2 3 4 5 6

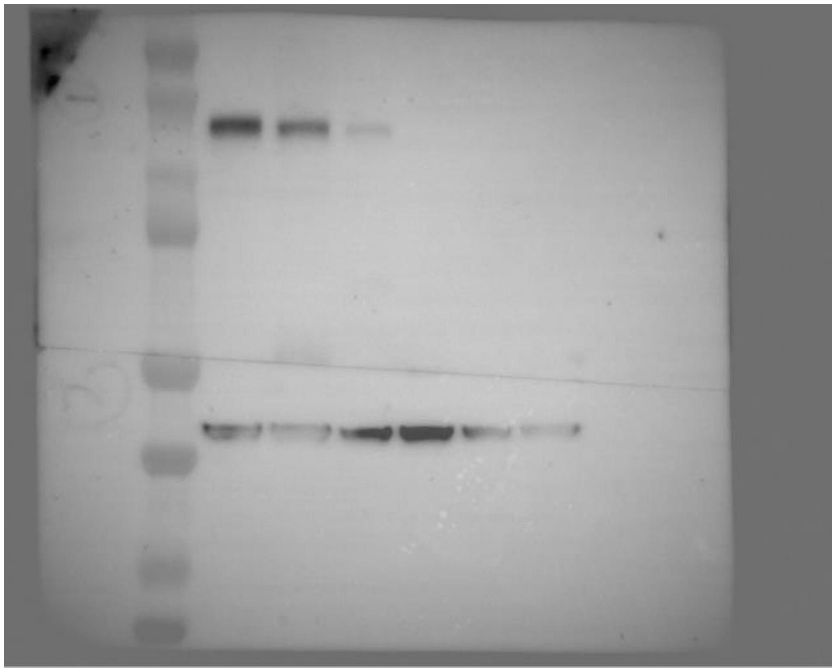

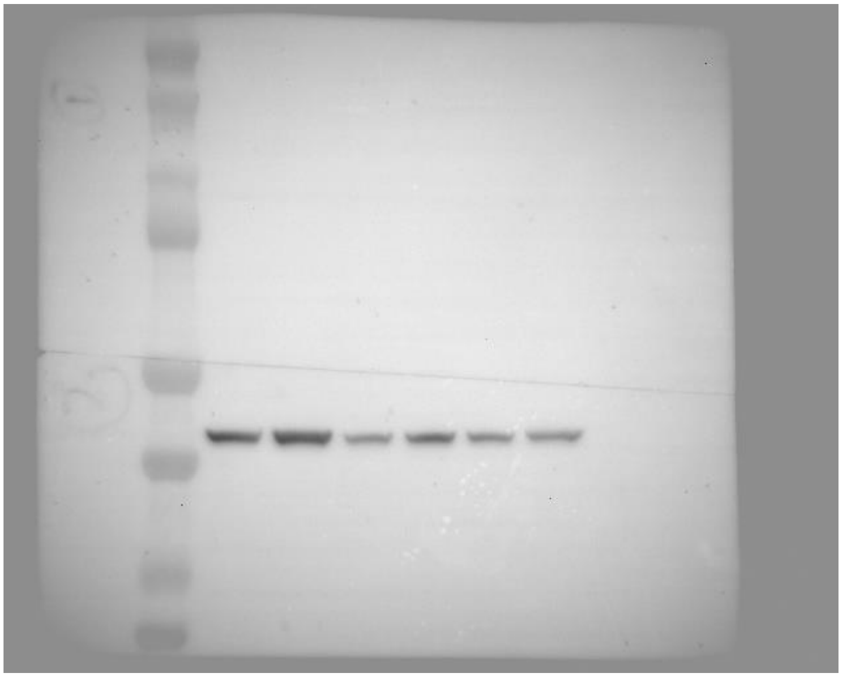

Marker 1 2 3 4 5 6

Blot Information

This blot was used for Fig 4A. The Top blot was chosen to show PTGFRN expression, and the bottom blot was used to show the actin loading.

It should be noted that this membrane was cut in order to incubate the two sections in different primary WB antibodies, with the upper half of the membrane being probed with anti-PTGFRN, and the bottom half of the membrane was probed for anti-Actin.

1= A431 40µg

2= DAOY 40µg

3= MSTO-211H 40µg

4= JEG-3 40µg

5= TOV-21G 40µg

6= MDA-MB-231 40µg

Images were captured using Chemiluminescent solution and UVP machine
